# Exploring diabetes-related stigma in adolescence: A critical review

**DOI:** 10.4102/phcfm.v17i1.5175

**Published:** 2025-12-06

**Authors:** Nadine Janneke, Elmari Deacon

**Affiliations:** 1 Community Psychosocial Research (Compres), Faculty of Health Sciences, North-West University, Potchefstroom, South Africa; 2 Optentia Research Unit, Faculty of Health Sciences, North-West University, Potchefstroom, South Africa

**Keywords:** adolescence, critical review, perceived stigma, stigma, T1DM

## Abstract

**Background:**

Stigma experiences challenge Type 1 Diabetes Mellitus (T1DM) adolescents. Such an impact causes complications in their self-management behaviour and identity formation.

**Aim:**

To critically synthesise, analyse, interpret and reflect on research regarding the experiences of T1DM adolescents and stigma through identifying the types of stigma experienced and the impact they have on T1DM adolescents.

**Method:**

Three scholarly databases were used to identify scientific data, which was subjected to a screening process using the Preferred Reporting Items for Systematic Reviews and Meta-Analyses (PRISMA) method of extraction and analysis. One hundred and nine articles were scanned, yet 14 eligible articles were included in the review. Using thematic analysis, the experiences of T1DM adolescents and stigma were consolidated, improving our understanding of the interactive nature of stigma.

**Results:**

T1DM adolescents experience social, enacted, internal and self-stigma. These experiences result in suboptimal T1DM self-management and the non-disclosure of a T1DM diagnosis. Negative effects associated with stigma experiences are linked to challenges in T1DM identity integration and decreased wellness. A diagram was developed to explain the continuous interactive nature of stigma.

**Conclusion:**

Stigma experiences may have a negative impact on adolescents in the absence of support structures and appraisal strategies.

**Contribution:**

Within a primary care setting, practitioners are empowered to comprehend the stigmas experienced by T1DM adolescents. Through this knowledge, adolescents may be educated to cope with such experiences without compromising their T1DM self-management or their psychosocial development. Academically, the model can assist future researchers in understanding the relationships that exist between stigmas while informing opportunities for interventions in curbing the effects of stigma.

## Introduction

Well-being in adolescence matters.^1^ Adolescent well-being has been identified as a key priority by the United Nations 2030 Agenda for Sustainable Development in the Global Strategy for women’s, children’s and adolescents’ health 2016–2030.^2^ Sustainable Development Goal (SDG) 3 is directed at the promotion of health and well-being in adolescence; it is also the first time that adolescents are included alongside children and women in the Global Strategy Goals (2). The Global Strategy for Women’s, Children’s and Adolescents’ Health 2016–2030 provides challenges specifically pertaining to adolescent health and well-being. (1) The leading causes of death in adolescent girls are suicide and complications during childbirth, (2) eighty per cent of adolescents are not physically active and (3) seventy per cent of preventable adult deaths because of non-communicable diseases have their root cause in adolescence.^2^

Adolescent development (at ages 12–18 years) remains a particularly vulnerable phase during which numerous cognitive, identity, emotional and social changes occur.^3,4^ The main psychological aim, during adolescence, is solidifying a self and social identity that is distant from parents while having a strong focus on feedback from social interaction and input from peers.^3,4^ In unison with the striving for autonomy, cognitive changes occur that intensify identity development. Self-regulation and long-term planning remain processes that will only fully develop in young adulthood, and it appears that adolescents base their decisions on cognitive evaluation and psychosocial determinants.^5^ The prefrontal cortex and the limbic system, known to inform social cognition, self-consciousness, planning and decision making, are still in development.^3,5^ In potentially rewarding situations, limbic system activation occurs and dominates the underdeveloped prefrontal cortex’s ability to exercise cognitive control.^3^ Scholars agree and elaborate that adolescents find it difficult to make informed decisions, as is evident in risky behaviour.^6^ Risky behaviour is rewarded by peer acceptance and can include using drugs, smoking, drinking and engaging in dangerous activities.^7^ Risk-taking does not always result in extreme behaviour but is an exploratory activity aimed at self-resolving uncertainties, which are necessary to achieve developmental goals, knowledge and wisdom.^8^

Adolescents living with Type 1 Diabetes Mellitus (T1DM) need to, in addition to developmental challenges, fulfil the role of disease manager and self-administrator of life-dependent treatment through self-injecting. Self-injecting is considered to be a highly stigmatised act yet forms part of the daily reality for patients living with T1DM.

Recent global statistics in the International Diabetes Federation (IDF) Atlas Report communicate that 1.52 million people, under the age of 20 years, live with T1DM.^9^ Studies indicate a peak age of T1DM onset between the ages of 10 years and 15 years.^10^ To complicate the number of diagnoses under the age of 20 years, researchers found that childhood-onset T1DM patients carry a greater risk of developing anxiety and stress-related disorders, thereby compromising well-being.^11^ For these reasons, adolescents who cope with diabetes, while navigating through adolescence, deserve special research attention.

Diabetes is a chronic metabolic condition that develops as a result of the destruction or dysfunction of pancreatic beta-cells, resulting in insufficient production of insulin^12,13,14^ and can be caused by environmental, physiological, genetic or behavioural factors.^15^ Various classifications of diabetes exist. The most diagnosed form of diabetes is type 2 diabetes mellitus (T2DM), followed by T1DM, which accounts for 8.75 million people as calculated in 2022.^9^

In T1DM, the body erroneously attacks itself, causing the pancreas to stop manufacturing insulin.^16^ Insulin is a vehicle that facilitates blood sugar entry into the cells, which is converted to energy.^17^ As blood sugar builds up in the bloodstream, it causes fatally elevated blood sugar levels.^18^ Type 1 diabetes mellitus patients require immediate insulin replacement therapy, which involves the daily self-administration of insulin by means of an insulin pen, syringe or the use of an insulin pump,^19^ daily glucose monitoring through finger pricks and lifestyle modifications that include dietary control.^10,20^ For adolescents with T1DM, navigating through identity formation, independence, autonomy and social acceptance, diabetes management is a particularly challenging task.

Adolescents commence self-managing T1DM. Scholars found conflicting evidence on how adolescents interpret the transition from the parental management of their diabetes to the self-management thereof.^20^ Results indicate a positive correlation between parents acting in a supportive and co-managerial role, while an over-involvement of parents leads to increased anger and feelings of lowered social competence. In a longitudinal study, tracking glycaemic control from adolescence to early adulthood. Researchers found fluctuations in glycaemic levels and concluded risk factors as being, firstly, adolescents who internalise problems rather than voicing concerns and secondly, social difficulties such as conflict with friends.^21^

Glycaemic levels indicate the amount of sugar in the bloodstream. Typical aetiological complications of diabetes mellitus include hypoglycaemia, diabetic ketoacidosis, hyperglycaemic neuropathy and hyperglycaemic diabetic coma.^13^ To avoid complications, patients are required to continuously self-monitor blood sugar levels.^22^ Complications are not exclusive to unstable glycaemic levels; T1DM patients also face mental health complications. Type 1 diabetes mellitus adolescents have been noted to have significantly increased general anxiety levels, which are two to three times higher than those of their healthy peers.^23^ General anxiety levels may be influenced by the psychological impact of identity-disease integration while confronting diabetes-related stigma, which is considerably problematic in younger patients aged 14–24 years.^23,24^

Stigma is the possession of idiosyncrasies of a discrediting or different nature and is considered to devaluate a person’s identity, may disqualify a person’s humanity or act as a source of resilience.^25^ Scholars agree and elaborate on stigma as a social, multidimensional and complex phenomenon,^26^ which is proven to have a negative effect on health-seeking behaviour and the emotional well-being of young people and adolescents.^27,28^

Stigma is noted to hold many variants, including social, enacted, perceived, internalised and self-stigma. Social stigma entails the negative perception of society on a particular group or individual possessing a distinguishing characteristic that separates them from what is considered ‘normal’.^29,30^ Enacted stigma relates to the discrediting behaviour and acts directed at the stigmatised person, which can include exclusion in everyday activities and social distancing.^26^ The effects of social exclusion and isolation in adolescence have a negative impact and are linked to poor academic performance, social difficulties and poor mental health outcomes, including aggressive behaviour, depression and social stress.^31^ Perceived stigma relates to how the recipient of stigma values, appraises and assesses a particular stigma as important or relevant.^26,30,32^ When perceived stigma is appraised, internalised stigma follows. Internalised stigma relates to the experiences of living with a stigma and involves feelings of shame, self-blame and guilt^26,29,33^ with wider mental health and well-being implications relating to depression and anxiety.^34^

Various studies confirm that enacted, perceived and internalised stigmas are linked to self-stigma.^26,29,30^ Self-stigma occurs when labels are internalised and behaviour is adapted to reinforce the stigma.^26,30^ Literature warns of stigma’s negative implications on health.^26^ Added to this, scholars concur and elaborate on the disabling effects of stigma on the treatment and management of a disease, the utilisation of healthcare services, disclosure of a health condition and the adherence to treatment plans.^29^ In a systematic review, a clear correlation between the use of injectables as a perpetuating factor of diabetes stigma as it mimics the use of illegal substances has been found.^29^

Adolescents who live with a chronic disease, such as T1DM, find it more challenging to manage the biological, psychological and psychosocial factors that face them, while self-managing their diabetes, independent of parents.^35,36^ Experiencing stigma can be detrimental at any age, yet lifelong well-being is founded in adolescence.^37^

Taking these aspects into consideration, the 2030 Agenda for Sustainable Goals^2^ clearly marks the health and well-being of adolescents as a key priority and includes, as a focus, the minimising of risk factors experienced in adolescence because of non-communicable diseases, leading to adult deaths. Type 1 diabetes mellitus is a non-communicable disease, which relies on effective self-management. Diabetes-related stigma is mainly associated with the very act of self-management and self-administering of treatment.^29^ Social experiences of stigma are noted to influence general anxiety levels of adolescents.^23,24^

### Aim

It is for these reasons, this critical review aims to critically synthesise, analyse, interpret and reflect on previous research regarding the experiences of T1DM adolescents and stigma to provide a holistic, integrated and in-depth perspective on the experiences when stigma, T1DM and adolescence intersect. Special attention was given to the types of stigmas experienced and how they influence T1DM adolescents. Available scientific literature regarding T1DM and adolescence is ample, yet limitations exist on stigma experiences and how they influence adolescents living with T1DM.

## Method

### Research approach

A critical review perfectly positions this research in its quest to analyse existing literature on the topic. It allowed for a substantial investigation of the findings of identified literature and the interpretation thereof, allowing for a summarised body of knowledge. The approach further paved the way to move beyond a mere description of current research to the construction of a hypothesis founded in the thorough investigation of existing literature.^38^ All data collection and review occurred in 2023.

### Search method

With the assistance of a North-West University (Potchefstroom Campus) faculty librarian, EBSCOhost (including Academic Search Complete, APA PsycINFO, APA PsycARTICLES and SocINDEX), Scopus and ScienceDirect databases were used to identify relevant academic literature. The keywords used to identify relevant literature were adolescence*, diabetes*, stigma, type 1 diabetes* and T1DM. Boolean operators AND and OR were used to further clarify the scope of articles. A total of 109 articles were identified. Eighteen duplicates were removed, and a further five articles were removed because of their inaccessibility, narrowing the total number of articles included for screening to 86.

### Inclusion and exclusion criteria

All searches were restricted to incorporate published and peer-reviewed articles dated 2013–2023, allowing for relevant and current data. The age range of inclusion was limited to adolescents aged 12–18 years, currently living with T1DM. Full-text journals and those who followed a qualitative, quantitative or mixed method approach have been included, while review articles were excluded. Conference proceedings have been excluded, while Doctor of Philosophy theses, Master’s dissertations, and mini dissertations were included. Lastly, only articles that have been published in English or those that have been translated before their publication have been included in the search approach.

### Critical appraisal of literature gathered

Two independent reviewers scanned the initial article titles, abstracts, purpose and aims to determine relevance based on how they correlate to the review question and the keywords as guided by the inclusion and exclusion criteria. The title, keywords and abstracts were scanned to determine compliance with these criteria. Fifty-three articles were excluded, bringing the total number of articles to be presented for a full text scan to a total of 33.

Both the primary and secondary reviewers independently and manually reviewed the full text of selected articles, subjecting them to in-and-exclusion criteria while assessing the soundness of their methodology. Special care was taken to obtain ethical approval and ensure that both ascent and consent were provided for the participation of adolescents with T1DM in the review. After the articles were scanned independently, the primary and secondary reviewers discussed their findings. Each article was discussed, and the relevance to the study was examined. In cases where the reviewers’ opinions differed, reviewers reverted to the full text of the articles, and a collective decision was made. Through the process, 19 articles were excluded leaving a total of 14 scientific and peer reviewed articles that possessed rigour.

**FIGURE 1:**
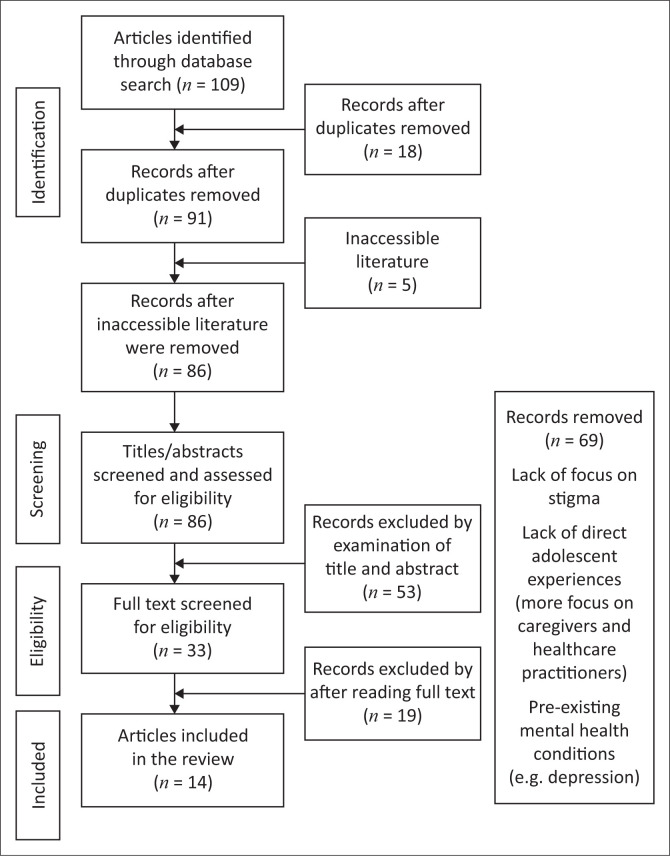
Preferred Reporting Items for Systematic Reviews and Meta-Analyses flow diagram showing article selection criteria.

Figure 1 visually depicts the Preferred Reporting Items for Systematic Reviews and Meta-Analyses (PRISMA) flow diagram, showing article selection criteria.

A total of 69 articles did not qualify for this review, and reasons for exclusion included a lack of focus on stigma and a lack of direct experiences of adolescents (more focus was given to experiences from parents, caregivers and healthcare practitioners). Further exclusions were based on adolescents with pre-existing mental health conditions, such as depression.

### Information synthesis

An in-depth understanding was obtained by both reviewers of the 14 articles identified for the review. Relevant information pertaining to the research question was extracted and tabulated in Microsoft Excel^©39^ and included the name of the author(s) and publication information (aim, research design and method), the country in which the study was conducted, the sample size and ages of participants, participant demographics, the main findings, stigma types, conclusions and limitations as published.

All relevant data were included in the synthesis for the purpose of transparency and relevance and to ensure that possible themes were not omitted. Such inclusions added to the transferability, reliability and validity of the findings. Specific focus was given to identifying the types of stigmas experienced, followed by an investigation into how stigma influences T1DM adolescents. For this to have crystallised, articles were read and re-read again, process notes were made and visually depicted to ensure an in-depth understanding of the subjective experiences of participants in context (for qualitative studies), while results from quantitative studies were listed and incorporated in the visual depiction of data. Mixed method studies were, initially, viewed in their entirety, whereafter qualitative and quantitative data were incorporated in the visual depiction.

### Data extraction

Following an in-depth understanding of data, data extraction was performed by the primary reviewer. The extracted data contain the title and author(s), country where the study was performed, participants and gender specifications, the research design and aim of each article, a summary of the main findings and the limitations of each article. Within the process of data extraction, care was taken to reflect on the aspects of each study, answering the current research question. Table 1 presents the extracted data.

**TABLE 1:** Data extraction table.

Number	Title and author(s)	Country and participant demographics	Research design and aim	Main findings	Limitations
1	‘They called me a terrorist’: Social and internalised stigma in Latino youth with Type 1 diabetes. Authors: G Crespo-Ramos; E Cumba-Aviles, M Quiles-Jimenez^40^	Puerto Rico, United States Adolescents: *n* = 65 Age: 12–17 years-old Female: *n* = 35 Male: *n* = 30	In this mixed-method study, we aimed to extend current knowledge on diabetes-related stigma among youth by exploring its prevalence and nature in a sample of T1DM Latino adolescents and examining its relationship with socio-demographic variables and depressive symptoms.	67.69% of participants reported the presence of at least one type of stigma (social stigma, internalised stigma). Adolescents living in urban zones and from larger families present a higher number of stigma experiences. The childhood depression scale indicated a significant association between stigma and depression, while anhedonia experiences were significantly related to the number of social stigma experiences. Of 96 stigma experiences noted, 56 related to social stigma and 40 to internalised stigma. The most frequent presentation of internalised stigma was noted as feeling different and self-stereotyping, which includes feelings of not being like others, perceived as a burden, weak, inferior and incapable of ‘normal’ life. Self-stereotyping consists of physical worries or problems accepting the T1DM diagnosis. Microaggression was the most common form of social stigma, which includes hostility, derogatory remarks, pity, joking, being looked at differently and being bullied. Subtle attacks are enforced by means of words and gestures (intentional or not).	All participants measured positively for depressive symptoms. (2) All participants were interviewed and assessed at a clinic, which indicates they have access to services. Because the study was conducted at a clinic, participants may have confused researchers with therapists. No assessment of media or macro-social structure as a stigma driver.
2	‘You Cannot Cure It, Just Control It’: Jamaican Adolescents Living With Diabetes. Authors: Moji Anderson, Marshall K. Tulloch-Reid^41^	Jamaica, West Indies Adolescents: *n* = 19 Mean age: 14 years-old Female: *n* = 14 Male: *n* = 5	This first qualitative study explores Jamaican adolescent T1DM experiences and stigma with the goal of providing guidance to healthcare professionals and policymakers on improving young people’s diabetes management.	Living with T1DM is ridiculed with exclusions and restrictions. T1DM report being excluded from social activities, such as sports and feeling constantly controlled by others (focusing on eating behaviour). Stigma is perpetuated through the self-management behaviours of T1DM adolescents. Adolescents have been called names, ridiculed, seen as evil and even mistaken for having HIV. Even the idea of having T1DM provokes fear through observed T1DM experiences. Adolescents engage in secrecy while trying to balance the ‘selves’ and living with T1DM. Support by parents is seen as overwhelming and controlling, and it suggests a lack of trust in adolescent abilities. Adolescents do, however, report neglecting self-management away from caregivers. Further, this hypervigilant, over-involvement by parents can increase public focus on T1DM while driving parent–adolescent conflict. Selective disclosure of T1DM helps fight self-stigmatisation.	None mentioned
3	Association of stigma, diabetes distress and self-efficacy with quality of life in adolescents with type 1 diabetes preparing to transition to adult care. Authors: Arij Soufi, Elise Mok, Mélanie Henderson, Kaberi Dasgupta, Elham Rahme, Meranda Nakhla^42^	Montreal, Canada Adolescents: *n* = 128 Age: 16–17 years-old Female: *n* = 74 Male: *n* = 54	In this quantitative study, the aim was to determine whether stigma, diabetes distress and self-efficacy are associated with quality of life (QoL) in adolescents with T1DM as they are preparing to transition to adult care.	Fifty nine per cent of the participants experienced diabetes-related stigma (perceived stigma) before transfer to adult care, and close to a quarter were identified as having diabetes distress, which is associated with a clinically significant lower QoL. Stigma has an impact on adolescents’ sense of identity, management of glycaemic levels, while lower QoL is linked to compromised mental health outcomes.	Casualty cannot be determined between psychosocial factors. Unmeasured confounders may have mediated associations made. Not all stigma items on the BDA scale were measured.
4	Developing a Personal and Social Identity With Type 1 Diabetes During Adolescence: A Hypothesis Generative Study. Authors: Persis V. Commissariat, Joslyn R. Kenowitz, Jeniece Trast, Rubina A. Heptulla, Jeffrey S. Gonzalez^43^	Bronx, New York, United States Adolescents: *n* = 40 Mean age: 16.5 years-old Female: *n* = 19 Male: *n* = 21	The purpose of this mixed-method research design study was to explore adolescent personal and social identity development with T1DM.	Life with T1DM is burdensome, triggered by the intensive daily self-management and balancing the responsibility of physical wellness with social life. T1DM leaves visible marks. Adolescents want to feel normal, yet the physical signs serve as a reminder that they are not normal. The visible signs of T1DM are, however, an indication of adherence to treatment plans. Despite the risks presented by stigma, positive experiences are noted when adolescents disclose their T1DM. Successful integration of T1DM into identity is imperative and increases self-management, while it decreases the effects of perceived and actual stigma. T1DM is described as being burdensome with inner conflicts between achieving goals versus self-management of T1DM, possible complications and negative mood changes. Participants further struggle with the incorporation of diabetes into their identities and ‘social selves’ while merely containing diabetes within limits. To contain T1DM, adolescents choose non-disclosure to avoid stigmatisation. Over-involvement of families and teachers feeds into them ‘not feeling normal’, giving birth to conflict. These acts endorse felt stigma. Positive peer relationships seem to buffer stigmatisation. Girls verbally share more of their T1DM experiences compared to boys.	To minimise the potential effects of biases, triangulation and audit trails were used. Parents were present during interviews, which could impact on results. Interviews were held while participants waited for an appointment at a medical facility, which could further influence findings
5	Diabetes Stigma Predicts Higher HbA1c Levels in Australian Adolescents With Type 1 Diabetes. Authors: Jesse A. Ingram, Jeneva L. Ohan, Keely Bebbington^44^	Perth, Australia Adolescents: *n* = 76 Mean age: 14.3 years-old Female: *n* = 40 Male: *n* = 36	This novel quantitative study’s aim was to explore the prevalence of diabetes stigma in adolescents with T1DM. The second aim was to explore whether adolescents’ experience of diabetes stigma is comparable to a previously published sample of adults. The third aim was to identify whether diabetes stigma predicted glycaemic control. It was hypothesised that both enacted and felt stigma would predict higher HbA1c levels.	98.7% of adolescents experienced at least one stigma type as listed on the stigma subscales (DSAS-1). Experiences included being treated differently, experiencing blame and judgement (unfair assumptions about capabilities, judged for eating behaviour, being irresponsible, causing own T1DM, microaggression) and identity concerns (self-conscious about self-management, non-disclosure because of fear, perception of injecting). Experiencing blame and judgment was of most concern, specifically through others making unfair assumptions regarding adolescent T1DM capabilities. Adolescents reported a higher degree of felt stigma compared to adults. Further, the hypothesis linking felt and enacted stigma and its impact on higher HbA1c levels is supported in this study, thereby confirming the link between felt stigma and compromised self-management. T1DM adolescents are more attentive to stigmatising behaviours from others. Adolescents report being rejected by friends, teachers and romantic partners and, as such, avoid social contact. Misconceptions regarding T1DM are associated with T2DM and were reported by 63.6% of participants. Adolescents report fear and worry of experiencing stigma because of uneducated peers.	DSAS-1 measure was not specifically designed for adolescents, and some stigma aspects may not be captured. Recruitment of T1DM adolescents remains challenging, as researchers aimed for a larger sample size, which could have increased the generalisability of the study.
6	Does Type 1 Diabetic Adolescents’ Fear of Stigmatisation Predict a Negative Perception of Insulin Treatment? Authors: Hamdiye Arda Sürücü, Gülbeyaz Baran Durmaz, Engin Turan^45^	Turkey Adolescents: *n* = 80 Age: 10–18 years-old Mean age: 13.3 years-old Female: *n* = 47 Male: *n* = 33	The purpose of this quantitative study was to investigate stigmatisation, socio-demographic and diabetes-related characteristics and parental characteristics as predictors of a negative perception of insulin treatment in adolescents with type 1 diabetes in Turkey.	75.5% of the sample did not disclose their T1DM to peers, 65% avoided injecting insulin in public places while 86.2% preferred to inject insulin while out of public eye. A significantly positive relationship was found between the negative perception of insulin treatment and stigmatisation, as well as between sharing T1DM status and private self-management. As stigmatisation increases, so does the fear of being rejected and treated differently by peers. A high level of social anxiety exists. Turkish cultural aspects contribute to these fears. Healthy Turkish men are seen as strong, while healthy women are seen as fertile. To avoid being seen as unhealthy, T1DM diagnosis and management are hidden from the public eye. T1DM adolescents in this study feel that they do not have an identity.	None mentioned
7	Institutional role conflict in the digital age: The case of diabetes management at school. Authors: Cassidy Puckett, Jenise C. Wong, Sloan Talbot, Hyojin Jennifer Min, Nora Chokr^46^	Northern California, US Adolescents: *n* = 19 Age: 11–14 years-old Female: *n* = 10 Male: *n* = 9	We investigate, in this qualitative study, different institutional roles in healthcare and schools, how these roles can conflict during day-to-day diabetes management in school and how youth with diabetes shoulder the burden of managing these conflicts.	The study found that school rules and norms interfere with T1DM self-management while enforcing and facilitating stigma. Learners are ‘restricted’ from managing T1DM in classrooms – even when devices are used. The beeping sound of devices or cell phones is said to disrupt classes, and examples are made from students, thereby increasing the stigma of ‘being different’. This example is followed by peers under the banner of ‘promoting school policies’. Similar experiences are communicated when participants refuse to eat food provided by the school because of dietary regulations.	A possible selection bias because of youth volunteering for the study, thus possibly drawing only on youth that is strongly opinionated. The sample is limited in size and demographics, and findings cannot be generalised to include all races or ethnicities and classes, especially those living in poverty. Families with higher socio-economic status may mediate these findings at school more successfully.
8	Lived Experiences of Newly Diagnosed Type 1 Diabetes Mellitus Children and Adolescents in Uganda. Authors: J. Nsamba, G. Nabirye, S. Hence, F. Drenos, E. Matthews^47^	Uganda, Africa Adolescents: *n* = 20 Age: 06–18 years-old Female: *n* = 12 Male: *n* = 8	This first Ugandan qualitative study explored the lived experiences, perceptions and strategies to cope with the news of T1DM diagnosis through the lens of Ugandan children and adolescents.	Newly diagnosed adolescents found it hard to come to terms with the diagnosis because of a pre-instilled fear of both the lethality of chronic diseases and their causes. Cultural superstitions of chronic diseases include being bewitched. Adolescents (before diagnosis) observed family members being diagnosed with ‘Sukaali’, which increased fear of diagnosis and increased death anxiety upon diagnosis. Participants face both positive and negative experiences from stress, anxiety and depression because of stigma, disease management and adverse reports. Stigmatisation from peers causes a loss of belonging that negatively impacts psychological well-being. T1DM adolescents are regarded as being sick. Peers avoid contact with T1DM friends and exclude them from social activities (play) because of false perceptions of T1DM contagiousness. Adolescents are socially, emotionally and psychologically affected by a T1DM diagnosis. Although a negative perception exists, some adolescents report receiving support from family and peers. Herbal medicines are promoted by families, which jeopardises T1DM treatment. Further misconceptions of T1DM relate to T1DM being an adult disease linked to early mortality. A clear lack of T1DM knowledge exists in Uganda, driving death anxiety.	This is an initial small study; thus results cannot be generalised to the population.
9	Living with type 1 diabetes is challenging for Zambian adolescents: qualitative data on stress, coping with stress and quality of care and life. Authors: Given Hapunda, Amina Abubakar, Fons van de Vijver, Frans Pouwer^48^	Zambia Adolescents: *n* = 10 Mean age: 15.3 years-old Female: *n* = 8 Male: *n* = 2	This qualitative study explored sources of stress, ways of coping with stress, perceived quality of care and life as experienced by Zambian adolescents living with type 1 diabetes (T1D).	Adolescents reported that they would not disclose their T1DM diagnosis as a major stressor because of a fear of discrimination. Adolescents feel different because of carrying self-management equipment with them, as well as about the visibility of self-management. Adolescents further report that they felt constantly monitored, cannot even be left alone and some family members give advice to stop their treatment. Stigmatisation and discrimination are common occurrences driven by both peers and society. Cultural beliefs exist that assume T1DM patients are not reproductively fit and thus romantic relationships with them should be avoided, especially if the T1DM patient is female. Males also faced challenges, yet more specifically, death anxiety. Further, very few of the Zambian languages have T1DM vocabulary, making it increasingly difficult to communicate about T1DM. Stigma is enacted through excluding patients from normal activities because of a fear of T1DM contagiousness. Misconceptions regarding T1DM are based on T1DM being a communicable disease comparable to HIV.	Because of the sample size, results cannot be generalised. Many opinions have been given by caregivers, and thus, a study focusing solely on adolescents’ experience would prove more fruitful.
10	Societal Norms and Conditions and Their Influence on Daily Life in Children With Type 1 Diabetes in the West Bank in Palestine. Authors: Kawther Elissa, Ewa-Lena Bratt, Åsa B. Axelsson, Salam Khatib, Carina Sparud-Lundin^49^	West Bank, Palestine, Middle East Adolescents: *n* = 10 Age: 08–18 years-old Female: *n* = 5 Male: *n* = 5	The aim of this qualitative study was to explore the experience of daily life in children with T1DM and their parents living in the Palestinian West Bank.	Cultural and social contexts largely influence the daily life of adolescents. Participants felt stigmatised because they are sick with an incurable disease. Fears of rejection and labelling are prominent in their lives, and thus, T1DM manages their disease in private or compromise treatment. This mismanagement allows them to be less different and more accepted by their peers. T1DM causes deep frustration. Fear and disgust are both feelings displayed towards adolescents with T1DM, impacting on the lack of disclosure. Women hold a negative self-image because of the effects of insulin and its visible markings.	A small sample size limits the findings. Potential translation errors may have been made, yet measures of forward and back translation have been adopted to minimise these errors.
11	Stigma and Its Association With Glycemic Control and Hypoglycemia in Adolescents and Young Adults With Type 1 Diabetes: Cross-Sectional Study. Authors: Anne-Sophie Brazeau, Meranda Nakhla, Michael Wright, Mélanie Henderson, Constadina Panagiotopoulos, Daniele Pacaud, Patricia Kearns, Elham Rahme, Deborah Da Costa, Kaberi Dasgupta^50^	Canada Adolescents: *n* = 380 Age: 14–18 years-old Female: *n* = 257 Male: *n* = 118 Gender fluid *n* = 5	The aim of this qualitative study was to estimate stigma prevalence in youth (aged 14–24 years) with type 1 diabetes and its associations with glycaemic control.	Sixty five per cent of participants reported the presence of stigma, with a clear link between poor glycaemic control, self-management avoidant behaviour and lower well-being because of the presence of stigma. Youth who reported experiencing stigma were three times more likely to have an haemoglobin A1c (Hb1Ac) of above 9%. Stigma was associated with a lower sense of well-being and less self-efficacy for diabetes management. Stigma experienced was observed to be slightly higher in young adulthood compared to adolescents, thereby establishing the importance of addressing stigma before transitioning to adult care. Stigma, by its primary definition, is observed higher proportion in girls than boys (69% versus 59%), confirming that girls feel more embarrassed by their T1DM status than boys.	Because of the cross-sectional nature of the study, causality cannot be proven. This large sample assisted in determining the presence of stigma, yet questions relating to the representativeness of the sample appear – those who experience stigma were more likely to have participated. Not all participants provided a capillary blood sample; however, the data collected were sufficient. Some participants may have provided fictitious answers.
12	The Challenges of Being Physically Active: A Qualitative Study of Young People With Type 1 Diabetes and Their Parents. Authors: Leanne Fried, Tarini Chetty, Donna Cross, Lauren Breen, Elizabeth Davis, Heather Roby, Tanyana Jackiewicz, Jennifer Nicholas, Tim Jones^51^	Australia Adolescents: *n* = 14 Age: 13–18 years-old Female: *n* = 8 Male: *n* = 6	We qualitatively investigated the challenges experienced by adolescents and young adults (AYA) with T1DM when physically active.	Stigma experienced by AYA, while physically active, relates to the carrying of their T1DM equipment, which visually distinguishes them from others. Further, adolescents report that they receive ‘special’ privileges while wanting to be treated like everybody else, and thus, they opt to partake in more individual sports rather than team sports. Team sports require disclosing T1DM status. Rules in team sports exclude T1DM adolescents, such as not being allowed to wear any equipment while participating.	The findings do not reflect on those AYA who are not physically active.
13	Understanding the Lived Experience of Children With Type 1 Diabetes in Kenya: Daily Routines and Adaptation Over Time. Authors: Tom Palmer, Cynthia Waliaula, Geordan Shannon, Francesco Salustri, Gulraj Grewal, Winnie Chelagat, Hannah M. Jennings, Jolene Skordis^52^	Kenya, East Africa Adolescents: *n* = 15 Mean age: 12.7 years-old Female: *n* = 8 Male: *n* = 7	In this qualitative study, the aim was to expand current understanding of the lived experiences of children living with T1DM in a cohort of children, carers and health workers in Kenya.	Although stigma is prevalent in both high- and low-income settings, a challenge is presented by cultural aspects such as a belief in witchcraft and a reliance on traditional medication, which leaves T1DM adolescents to manage their disease in secrecy and in fear of disclosing symptoms (pre-diagnosis). T1DM adolescents are sometimes treated as ‘doomed’, ‘bewitched’ or ‘cursed’, evident through the labelling behaviour of their peers. The result of these acts is non-disclosure. Labelling behaviour extends to other systems – some schools are reported to refuse admission of T1DM learners. Further challenges include limitations in T1DM management at schools where teachers are described as uncooperative, peers’ lack of understanding and technical aspects pertaining to insulin storage – T1DM adolescents fear that their diabetic equipment will be stolen at school. T1DM misconceptions relate to beliefs in witchcraft, confusion that exists between knowledge of T1DM and T2DM (a lifestyle disease that does not affect children), the reliability of traditional medicines as a replacement for Western medicines, as well as limited knowledge of non-communicable and communicable diseases (only HIV knowledge is common). T1DM adolescents have a deep desire to fit in.	All participants have access to care, thus do not reflect the opinions of those without access. Photo diary information was not discussed with participants, which could have provided rich data. Many of the photos of participant diaries included faces of others who have not consented to the study. +E18
14	Illness Experiences of Adolescents with Type 1 Diabetes. Author: Ji Eun Kim^53^	Korea Adolescents: *n* = 12 Age: 13–19 years-old Female: *n* = 7 Male: *n* = 5	The purpose of this qualitative study was to develop a substantive theory that can explain the experience process by identifying the meaning that adolescents with T1DM give to their illness experiences and the social context that causes them.	Adolescents view having T1DM as a flaw in their identity. This ‘forced identity’ is difficult to incorporate into their own identity. They feel overwhelmed and surrounded by stigma and discrimination. Stigma alienates adolescents; even acts of sympathy and caring towards T1DM adolescents are perceived as stigmatising acts. Overwhelmed feelings further include having limited social activity and being dependent on and judged by parents. Adolescents struggled to cope with stigma and reported being hypervigilant towards stigma experiences. Attempts to protect oneself from stigma include social withdrawal and isolation. In order to accept T1DM, adolescents need to work through self-banishment towards acceptance, yet stigma infiltrates all aspects of life and presents humiliation that adolescents are unable to confront. Reports that T1DM is caused by an unhealthy lifestyle are also present.	The study presents holistic findings that limit in-depth views on focussed areas as identified. Being a small study, trends beyond cultural boundaries have not been explored. Findings cannot be generalised.

T1DM, type 1 diabetes mellitus; T2DM, type 2 diabetes mellitus; HIV, human immunodeficiency virus; BDA, barriers to diabetes adherence; Hb1Ac, haemoglobin A1c.

## Review findings

In total, 14 publications were included in this critical review, three from the United States, three from Africa, two from Canada, two from Australia, one from Turkey, one from Korea, one from Jamaica and one from Israel, providing a wide scope of data through various contexts and cultures. Of the 14 articles, nine produced qualitative data, three focused on quantitative methods and two utilised mixed method approaches. Of the 14 articles, three were pioneering studies focusing on the experiences of T1DM adolescents and stigma in their specific areas or countries, Jamaica, Perth and Uganda. Combined, these studies allowed access to the experiences of 888 adolescents, of which 544 were female, 393 male and 5 gender fluid individuals.

### Types of stigma

Four stigma types were identified in all 14 articles of the review, namely enacted stigma, internalised stigma, social stigma and self-stigma. The types of stigmas and the emerging themes, as identified in the review, are illustrated in Table 2.

**TABLE 2:** Themes emerging from reviewed articles.

Stigma type	Common experiences
Enacted	Experiences at school Experiences at home Microaggression
Internalised	Non-disclosure of Type 1 diabetes mellitus status
Social	Social trypanophobia Misconceptions about Type 1 diabetes mellitus
Self	Identity integration concerns

### Enacted stigma

The most common form of stigma experienced by T1DM adolescents is enacted stigma. Enacted stigma refers to the discrediting behaviour, verbally or non-verbally, intentionally or non-intentionally, directed at a stigmatised person.^26^ Through analysis of the articles, major themes emerged as being experiences at school, experiences at home, followed by microaggressions.

The effects of enacted stigma can be significantly linked to compromised T1DM self-management and higher haemoglobin A1c (Hb1Ac) levels, having serious consequences on health and a negative impact on psychological well-being.^44,47^

#### Experiences at school

Stigma experiences at school are evident in the exclusion of T1DM adolescents from participating in sports or social activities.^41,44,46,47,48,49,51,52,53^ Adolescents report this exclusion to be linked to the visibility of T1DM management that distinguishes them from peers.^41^ Type 1 diabetes mellitus adolescents share a key distinguishing factor, differentiating them from their healthy peers; they have the responsibility of self-management, whereas most of their peers have no responsibilities.^43^ Considering that adolescents spend most of their time at school, these findings are worrisome.

On the school grounds, stigma is experienced through the close monitoring and reprimanding behaviour of teachers at school, specifically related to self-management.^14,46,48^ T1DM adolescent learners report being restricted to publicly self-manage during school hours. Adolescents are required to manage their sugar levels under the watchful eye of a school nurse.^46^ Because of a fear of missing class, learners manage their T1DM from ‘behind the classroom in a bush’, making them more vulnerable to adverse stigma reactions and labelling from peers (p. 5).^46^ Many learners are required to carry self-management devices. When devices alert them of unstable levels during class times or tests, teachers get annoyed and publicly reprimand learners for disrupting the class, categorising them as rulebreakers.^46,52^ As one adolescent describes, ‘Teachers do not get it too. It’s just hard, you’d rather do everything secretly’ (p. 150).^52^ Secrecy in self-management, coupled with the public reprimanding and monitoring by teachers, drives the stigma that T1DM adolescents are different from their peers and even incompetent in caring for themselves. Non-disclosure and compromised self-management solve the fear of such stigma.^44,46,47,53^

On the sports field, school policies prohibit the carrying of equipment, such as insulin pumps, while engaged in team sports, which physically distinguishes T1DM adolescents as different.^51,53^ When T1DM adolescents do participate, they are forced to disclose their T1DM status and report receiving unwanted special privileges.^14,51^

Added to this, teachers fear the effects of hypoglycaemia. Fearing their inability to assist in these situations, they decide to exclude T1DM adolescents from teams.^49^ Being active increases the need for public T1DM self-management. Here too, adolescents report how people’s judgemental, negative reactions to their self-management highlight their visibility next to the sports field.^49,51^ To avoid all these complications and exclusions, T1DM adolescents opt to participate in individual sports, further limiting their opportunities for developing healthy peer relationships.^51^

#### Experiences at home

Stigma experiences at home revolve around overprotective parents who mistrust the abilities of their T1DM adolescent to effectively self-manage. Such parents are reportedly overprotective and controlling to the extent that they create dependent adolescents.^14,43,46,47,51,53^ Constant supervision from parental figures enforces the perception that adolescents are different from their peers, even vulnerable.

While most articles report on the overprotection of T1DM adolescents by parents, an article from Kenya explains how adolescents of uninvolved parents successfully manage T1DM, yet, in spite of successful self-management, the desire to be part of, and fit into a social group remains.^52^

Enacted stigma at home is clearly evident in parental reluctance to allow adolescents the opportunity to become independent through learning to self-manage their T1DM. Acts such as these set the stage on which T1DM adolescents feel less worthy and increase their vulnerability to experiences of microaggression.

#### Microaggression

Microaggressions are verbal, non-verbal or subtle acts directed at T1DM adolescents to discredit them.^40^ Physical acts are described to include being publicly ridiculed, called names, regarded as sick, labelled, cursed, bewitched, doomed or sometimes mistaken for having HIV^41,47,48,52^ and extend to acts where T1DM adolescents’ equipment is being stolen at school, while no provision is being made for the storage of insulin.^52^

At school, peers observe how teachers act negatively towards T1DM adolescents and follow their example by acting with microaggression, under the banner of ‘promoting school polices’.^41,46^ T1DM adolescents are observed by teachers and reprimanded when they eat something sugary; these acts are extended by peers calling T1DM adolescents names such as ‘sugar mamma’, ‘patient’ or ‘you soon dead’.^41,47,49,53^ Acts of microaggression further enhance the stigma that T1DM adolescents caused their own T1DM through their unhealthy diet.^53^ Discrediting acts by teachers are fueling the use of digital T1DM management tools.^46^ As mentioned earlier, many T1DM adolescents use devices and cell phones to monitor sugar levels. When these devices signal in class or during tests, teachers are immediately frustrated and irritated and reprimand the T1DM adolescent. Peers adapt and respond with laughter, ridiculing the T1DM adolescent. Fearing these scenario’s T1DM adolescents turn their devices off, thus compromising self-management.^46^

### Internalised stigma

Internalised stigma relates to the experiences of living with a stigma and involves feelings of shame, self-blame, guilt, being a burden to others, weak, inferior and abnormal,^26,29,33^ leading to wider negative mental health and well-being outcomes, such as depression and anxiety.^34^ Internalised stigma has been identified as the second most frequent stigma type experienced by T1DM adolescents. Through thematic analysis, such experiences can be consolidated under two sub-themes, namely the non-disclosure of T1DM status and feeling different.

#### Non-disclosure of Type 1 diabetes mellitus status

The perception of stigma, and the evaluation thereof as important, is significantly linked to the decision to disclose T1DM status.^41,45^ Most of the articles describe how T1DM adolescents fear disclosing their status because of the presence of stigma, fear of rejection and the humiliation of public self-management.^41,43,44,45,47,48,49,52,53^ Injecting in public is a constant worry that increases social visibility and influences what peers may think.^44^ Non-disclosure is a safer alternative in protecting the T1DM adolescent from social adversities, as already felt or observed through enacted stigma.

Those brave enough to disclose their T1DM reap the benefits and report positive experiences through the continuous support of friends and family, receiving assistance from peers in difficult times and a reduction of stigma experiences.^41,43,47^ Various researchers agree, and results indicate a significant link between adolescents sharing their T1DM status and reduced stigma experiences, the positive perception of insulin treatment and private self-management.^45^

### Social stigma

Social stigma ranked third most frequent. Themes identified within social stigma are trypanophobia, misconceptions about T1DM and culturally driven stigma. Social stigma sets the stage for the existence of all types of stigmas. The effect of social stigma leads to stigma-avoidant behaviour, such as managing T1DM in secret and out of the public eye.^45^ Authors have demonstrated a significant relationship between stigma and the negative perception of insulin treatment, disclosing T1DM status and self-management.

#### Social trypanophobia

The management of T1DM involves testing sugar levels via a finger prick and injecting insulin to control HbA1c levels.^10,20^ Nine articles address the public injecting of insulin as used in T1DM self-management as a source of stigma.^41,43,44,45,47,48,49,50,52^ Adolescents describe the difficulties of self-management because of the stigmatised view of needles, while receiving negative evaluations from peers assuming T1DM adolescents are taking drugs, inducing an abortion or have HIV.^41,43,44,49^ Stigmatised views are not only confined to the act of injecting but also focus on the visible signs at the sites of injection that deform adolescent’s body image.^43,49^

#### Misconceptions about type 1 diabetes mellitus

Common social misconceptions regarding T1DM are based on a lack of T1DM disease knowledge and on cultural superstitions. The most occurring lack of T1DM knowledge is that this non-communicable disease is, in fact, contagious and that its root cause is ascribed to maintaining an unhealthy lifestyle, thus explaining the misinterpretation between T1DM and T2DM.^44,45,47,48,52,53^ Resulting from this, T1DM adolescents are excluded from opportunities, such as participating in sports or joining social events.^41,44,46,47,48,49,51,52,53^ Adolescents resolve to isolation and risk T1DM complications rather than facing the wrath of false beliefs associated with T1DM.^44,45,47,48,52,53^

A last yet noteworthy and frequently occurring theme under social stigma is found in cultural superstitions derived from articles in Jordan and Africa, where T1DM is believed to be a punishment for previous family or individual sins or being the victim of witchcraft.^45,47,48,52^ Such experiences affect not only adolescents but also their families, leading to secrecy, overprotection, fear and avoidant behaviour from the entire familial system.^45,47,48,52^

Social stigma drivers in Turkey view male T1DM adolescents as weak, while females are considered less fertile.^45^ In both Kenya and Uganda, female T1DM adolescents have a similar fate and are considered less fertile plus incapable of caring for large households.^47,52^ Ugandan T1DM adolescents are further considered bewitched with ‘*Sukaali*’, which is linked to early mortality.^47^ The extent to which stigma infiltrates the life of Zambian T1DM adolescents incorporates linguistic issues. Zambian adolescents share their frustration of not being able to describe their experiences regarding T1DM in words, as seldom words exist to explain T1DM in local languages.^48^

Complications of social stigma have no boundaries, as they dictate the accepted norms of society. Type 1 diabetes mellitus adolescents have to cope with their diagnosis while adapting to the responsibility of self-management. While this remains a difficult task in a stable and supportive environment, imagine the impact of attempts to incorporate T1DM into identity in a hostile environment, ridden with stigma.

### Self-stigma

Self-stigma occurs when labels are internalised and behaviour is adapted to reinforce the stigma.^26,30^ Crespo-Ramos^40^ and colleagues define self-stigma as a sub-category of internalised stigma, which incorporates any stigma directed towards the self, being either physical, spiritual, psychological or emotional and can occur in combination.^40^ Self-stigma within these articles occurs when T1DM is not incorporated into identity, which causes stigma to infiltrate every aspect of the self and allows it to direct and determine behaviour.^40,41,43,45,48,49,53^

#### Identity integration concerns

Incorporating T1DM into identity appeared as the most concerning theme under self-stigma. Adolescents experience inner conflict because of T1DM, see themselves as less autonomous, differentiated from what is expected from them both culturally and according to gender roles, as well as being incapable of living a normal life.^40,41,43,45,48,49,53^ Adolescents with T1DM struggle to integrate T1DM into their identity, hereby denying their T1DM status while simultaneously not practising optimal self-care.^40,41,43,45,53^ This form of malintegration leads to a struggle to balance ‘the self’ with T1DM.^41^ Stigma hinders T1DM identity integration as described through the ‘forced identity’ that T1DM adolescents are living with.^53^ Fourteen articles describe how this ‘forced identity’ hinders the ability to fit in, to feel normal and to accept the self as autonomous, even as a part of a cultural group.^40,42,43,44,45,46,48,49,50,51,52,53^

Adolescents who incorporated T1DM into their identity and self-concept viewed T1DM as a part of their new selves and viewed self-management as a challenge rather than a threat. For these individuals, stigma experiences are seen as opportunities to educate peers about their illness.^43^ Adolescents who incorporated their T1DM did not allow the negative reactions of peers to affect their self-management; they took direct, active roles in addressing diabetes in social situations, shared their diabetes status and educated others about their illness.^43^

The influence of stigma experiences was observed in all studies, with negative experiences of stigma found to cause a lower quality of life (QoL), which impacts identity development and integration.^42^ Quantitative studies reported 59% to 98.7% of adolescents living with at least one stigma experience.^40,42,44,45,50^ Articles reviewed noted how negative stigma experiences affect physical, emotional and psychological well-being.^45,50^ Negative stigma experiences have dire results,^40^ where all their participants (65 adolescents) presented with the effects of self-stigma in the form of depressive symptoms. The findings further indicated a significant link between social stigma and experiences of anhedonia. Scholars who conducted studies in 2018 found similar results linking negative experiences of stigma to poor glycaemic control, self-management avoidant behaviour and lower physical, emotional and psychological well-being leading to the development of a fear of insulin treatment.^45,50^ A link has been established between enacted and felt stigma impact on higher HbA1c levels, which again compromises self-management.^44^ Adolescents who experience stigma are three times more likely to have an HbA1c of above 9%.^50^

## Discussion

This critical review sets out to analyse and synthesise the most updated research available on how adolescents with T1DM experience stigma. Two sub-questions guided the research by, firstly, identifying the types of stigmas that impact T1DM adolescents and, secondly, to consolidate how stigma influences adolescents living with T1DM.

Through this review, it can be inferred that enacted, internalised, social and self-stigma is experienced by most adolescents. Stigma leads to non-disclosure of T1DM status.^41,43,44,45,47,48,49,52,53^ Stigma impacts negatively on T1DM self-management.^41,42,44,45,46,47,48,49,50,52^ Stigma further influences healthy identity development, evident in the adolescent’s struggles to integrate T1DM into their identity.^40,41,43,45,48,49,53^ The major identified effect of stigma experiences is decreased well-being.^40,41,42,43,45,48,49,53^

Within any society, there are social norms and values that should be adhered to gain social acceptance. Behaviour and characteristics that contradict this norm can be described as social stigma.^29,30^ Social stigma is evident throughout the review in the discrediting manner in which T1DM adolescents are treated when attempting to publicly self-manage. Social stigma is further cradled through cultural superstitions, driving the enforcement of social norms and the avoidance of disease.^32^ The current review concurs with this view, visualising both the accepted social norm in Western and Arab-African societies, while linking disease avoidance through exclusion. Having identified common social misconceptions and cultural beliefs related to T1DM, it could be suggested that interventions aimed at public re-education should be effective. Researchers disagree and reiterate that social stigma or stereotypes are resistant to change.^32^

Social stigma fosters an environment conducive to the perpetuation of other forms of stigma, such as enacted stigma.

Enacted stigma unfolds when discrediting behaviour is directed at the T1DM adolescent who possesses the condition that society fears and discredits.^26^ In the review, this interactionist category of stigma was the most common type of stigma experienced by T1DM adolescents. Type 1 diabetes mellitus adolescents are excluded and discredited through verbal or non-verbal comments, they are bullied, excluded from school, social and sports activities in fear of being contagious or in fear of compromising their health.

Up to this point, the review produced findings on both social stigma and enacted stigma. Literature defines perceived stigma in contradicting ways. Various definitions of perceived stigma exist. Perceived stigma is the belief that individuals hold about others’ attitudes towards an illness,^54^ while another relates it as the awareness created by a stigmatised individual of prejudice, discrimination and stereotyping directed at them.^55^ Concerns are raised in this review about the appropriate understanding or definition of perceived stigma and if, in fact, it is a type of stigma.

**FIGURE 2:**
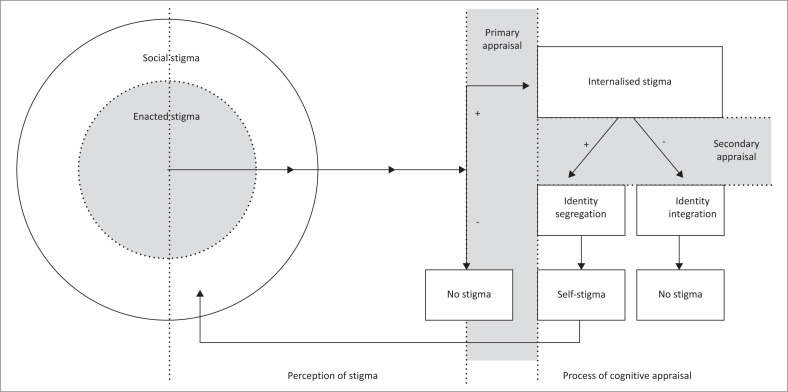
The interactive nature of stigma.

From the identified literature, a clear structure of the interactive nature of stigma emerged (Figure 2), clearly indicating the function of perception and the process of cognitive appraisal from social and enacted stigma to internalised and self-stigma.

In this review, and as illustrated in Figure 2, perceived stigma is found to be a process of the perception and cognitive appraisal of stigma rather than a stigma type. When an individual experiences either social or enacted stigma, it is channelled through a primary and secondary cognitive appraisal process. This process assigns value to the experience of the stigma. If the stigma is encountered and appraised as negative, no stigma is felt by the individual; however, if the individual assigns value to the stigma and primarily appraises it as positive, it results in internalised stigma.

Internalised stigma relates to the experiences of living with a stigma and involves feelings of shame, self-blame and guilt.^26,29,33^ In this review, internalised stigma is the second most prominent type of stigma experienced by T1DM adolescents. Through internalised stigma, T1DM adolescents appraise rejection and humiliation as effects of public self-management or the sharing of their T1DM status.^40,41,43,44,45,46,47,48,49,50,51,52,53^

This internalised stigma appraisal leads to a constant internal battle of self-awareness amplified by the signs of T1DM and its successful management. Literature concurs with the findings in this review, adding that internalised stigma experiences negatively impact well-being and wider mental health.^34^ Internalised stigma predisposes adolescents to hypervigilance for stigma experiences, leading to the appraisal of non-threatening acts as threatening, which inflates the experiences of enacted stigma. Similar experiences were found in studies surrounding sexual-minority groups, where hypervigilance to stigmatised acts placed individuals at risk for internalising their problems, potentially leading to anxiety and depression.^56^ Limited data are available on the function of hypervigilance to stigma experiences of T1DM adolescents. This novel finding could be an avenue to explore for future researchers.

Following internalised stigma, a secondary cognitive appraisal occurs, this time resulting in the decision to incorporate the stigma into self-identity or to reject the stigma. Type 1 diabetes mellitus adolescents who decline to incorporate stigma experiences choose to incorporate T1DM into their identity. Through the review, it was evident that those adolescents who negatively appraise stigma in the secondary cognitive appraisal process found it easier to incorporate T1DM into their identity, which resulted in minimal or no experiences of stigma, and reported taking an active stance in their self-management behaviour while disclosing their T1DM status and even educating peers about their condition.^41,43,47^ The opposite process is also true; findings indicated that adolescents who go through the stage of secondary cognitive appraisal and found truth value in the stigma refused to incorporate T1DM into their identity, leading to self-stigma.^40,42,43,44,45,46,48,49,50,51,52,53^

By definition, self-stigma occurs when labels are internalised and behaviour is adapted to reinforce the stigma.^26,30^ As stated, T1DM adolescents who self-stigmatise incorporate T1DM stigma into their identity, resulting in suboptimal and secretive self-management. Type 1 diabetes mellitus adolescents describe themselves as holding a ‘forced identity’ or as having no identity. This ‘forced identity’ has negative consequences on the ability to form an autonomous self. Self-stigma leads to isolation. Secrecy leads to self-selected exclusion from social activities as explained by participants opting to only participate in individual sports or even in some saying that they choose to be alone. The effect of selective exclusion impacts negatively on self-esteem and the development of a personal and social identity and limits opportunities for building resilience. These findings are consistent with research on the effects of secrecy and stigma.^57^

Lastly, secretive self-management reinforces the negative perception the public has on injecting, linking self-management to illegal drug abuse, consistent with the definition of self-stigma, stating that self-stigma reinforces social stigma. This finding completes Figure 2 on the interactive nature of stigma, illustrating how the experience of stigma is continuous unless interventions are aimed at challenging the processes of perception and cognitive appraisal of stigma.

To summarise, experiences of stigma by T1DM adolescents are vast and extend from their social world to their deepest core. Stigma not only influences the world that T1DM adolescents live in, but it also negatively impacts their ability to become healthy, confident, independent, contributing members of society. In applying the presented Figure 2 to future studies, reviewers hope that clarity can be given to the experiences of T1DM adolescents and others living with stigma, in an effort to curb stigma and the perception and appraisal of such stigma.

## Limitations

This review study was based on the results and discussions of 14 identified articles. In total, these articles provided access to the experiences of 883 adolescents from 14 countries. The diverse geographical spread of the article provides a thorough overview of the current research on T1DM, adolescents and stigma but cannot be generalised to a global representation. Further, some articles incorporated a large sample of participants, while others consisted of minute samples, and thus are not representative of all T1DM adolescent experiences per country, yet provide good baseline information.

### Suggestions for future research and interventions

Future research surrounding the clinical impact of stigma on T1DM self-management is needed urgently. Diabetologists or endocrinologists are invited to drive research on the clinical impact of stigma experiences on T1DM self-management and well-being. Interventions to extinguish the impact of stigma experiences for T1DM adolescents should be designed and applied in healthcare policy, within assistance or management programmes and within intervention programmes. (1) Type 1 diabetes mellitus assistance or management programmes should be aimed at educating parents through developmental transition periods, such as from childhood to adolescence. Parents need to be empowered with effective skills to hand over diabetes management to adolescents. (2) Intervention programmes assisting adolescents to integrate T1DM into identity should be designed and implemented as a matter of urgency. Such interventions will foster resilience in T1DM adolescents, which will allow them to appraise stigma as frivolous. (3) Research needs to be expanded on the stigma experiences of T1DM adolescents, focusing on the perception and cognitive appraisal of T1DM stigma.

## Conclusion

In this review, a thorough consolidation and synthesis of literature produced in-depth insight into the types of stigmas experienced by T1DM adolescents. Through the process of dissecting the stigma types, patterns of interaction between stigmas were observed, and a diagram was developed to illustrate the interactive nature of stigma. The impact of stigma experiences on T1DM adolescents where consolidated under suboptimal T1DM self-management, the promotion of secretive or non-disclosing behaviour and a lack of T1DM identity integration. The impact of stigma on this population has severe consequences for physical and mental health, well-being and normal adolescent development.

Limited scientific evidence is available on understanding T1DM stigma experiences globally, and a challenge is extended to fellow researchers to pursue such research.
